# The impact of a culturally adapted lifestyle intervention on the glycaemic profile of mothers with GDM one year after delivery – a community-based, cluster randomized trial in Sri Lanka

**DOI:** 10.1186/s12902-024-01643-z

**Published:** 2024-07-08

**Authors:** Thamudi D. Sundarapperuma, Prasad Katulanda, Champa J. Wijesinghe, Priyadarshika Hettiarachchi, Sudharshani Wasalathanthri

**Affiliations:** 1 Department of Nursing, Faculty of Allied Health Sciences, University of Ruhuna, Galle, 80000 Sri Lanka; 2 Department of Clinical Medicine, Faculty of Medicine, University of Colombo, Colombo, 00800 Sri Lanka; 3 Department of Community Medicine, Faculty of Medicine, University of Ruhuna, Galle, 80000 Sri Lanka; 4 Department of Physiology, Faculty of Medical Sciences, University of Sri Jayewardenepura, Nugegoda, 11222 Sri Lanka; 5 Department of Physiology, Faculty of Medicine, University of Colombo, Colombo, 00800 Sri Lanka

**Keywords:** Gestational diabetes mellitus, Insulin resistance, HbA1c, HOMA-ir lifestyle intervention, Postpartum women

## Abstract

**Background:**

A woman with a history of GDM has a high risk of developing type two diabetes (T2DM) in her future life. Lifestyle modifications are known to attenuate the progression of GDM to T2DM. Therefore, the aim of this study was to assess the impact of a simple, cost effective, culturally acceptable lifestyle intervention programme on the trajectory towards T2DM in women with a history of GDM.

**Methods:**

This cluster randomized trial was conducted in 100 postpartum women in three selected districts of Sri Lanka. The subjects were divided into intervention (*n* = 50) and control groups (*n* = 50) by cluster randomization method. A culturally adapted protocol (comprised of dietary and physical activity modifications) was administered to the intervention group. The glycemic profile was assessed using fasting and 2-hour post-OGTT plasma glucose and HbA1c, and insulin resistance by HOMA-IR at baseline and after one year of intervention.

**Results:**

The mean age (SD) of the subjects in the intervention and control groups were 33.0 (5.1) and 34.3 (6.5) years respectively. All glycemic and insulin resistance parameters (i.e. Fasting plasma glucose- FPG, 2-hour post-OGTT plasma glucose, HbA1c and HOMA-ir) were comparable (*p* > 0.05) between the two groups at baseline. FPG, 2 h post OGTT, HbA1c and HOMA-ir values between intervention vs. control (p) at 12 months were 87.3 vs. 123.2 (< 0.01); 106.5 vs. 156.1 (0.01); 5.3 vs. 6.8 (< 0.01) and 0.9 vs. 2.3 (< 0.01) respectively. All glycemic parameters showed a significant reduction in the intervention group at 12 months compared to baseline. In contrast, the control group showed a significant increase in FPG, 2-hour post-OGTT plasma glucose and HbA1c at 12 months compared to baseline. In multiple linear regression model adjusted for age, parity and family history, the control group showed an approximately 33 times risk of developing insulin resistance compared to the intervention group.

**Conclusion:**

The culturally acceptable and individualized lifestyle intervention was able to produce remarkable reductions in glycaemic and insulin resistance parameters among postpartum women with a history of GDM.

**Trial registration:**

Ethical clearance was obtained from the Ethics Review Committee of the University of Sri Jayewardenepura, Sri Lanka (ERC 52/14), Sri Lanka Clinical trial registration number Sri Lanka Clinical Trials Registry (SLCTR/2015/021 date 25.09.2015).

## Introduction

Gestational Diabetes Mellitus (GDM) is a global health concern which is common among Asian populations [1]. A woman with a history of GDM is reported to have a 7-fold relative risk of developing type 2 diabetes mellitus (T2DM) and a 2 to 5-fold relative risk of metabolic syndrome compared to a woman with a normoglycemic pregnancy [2]. Moreover, a Sri Lankan study has reported a 10-fold higher risk of developing T2DM in women with prior GDM compared to women without GDM during a 10-year follow-up period [3].

GDM is defined as carbohydrate intolerance resulting in hyperglycemia, with onset or first recognition during pregnancy [4]. According to the International Association of Diabetes and Pregnancy Study Groups Consensus Panel guidelines, the diagnosis of GDM is made with a fasting plasma glucose of ≥ 0.92 g/L (5.1 mmol/L) and/or 1-hour plasma glucose of ≥ 1.80 g/L (10.0 mmol/L) and/or a 2-hour plasma glucose of ≥ 1.53 g/L (8.5 mmol/L) [5]. GDM is considered a state of pre-diabetes where there is glucose intolerance due to deficiency of insulin secretion or chronic insulin resistance at cellular level which probably would have occurred even before the pregnancy [6, 7]. A prospective cohort study has shown that women with GDM in a previous pregnancy had a faster deterioration of β-cell secretory capacity and insulin sensitivity at a subsequent pregnancy compared to women without a GDM history [8]. Homeostatic model assessment (HOMA) which is the product of fasting plasma glucose and fasting plasma insulin [9] is a valuable indicator that has been used extensively in epidemiological studies of various ethnic origins to assess β-cell function and insulin resistance [10].

Lifestyle interventions delay or prevent the development of T2DM in women with a history of GDM [11]. Diabetes Prevention Programme research group has reported a 34% reduction in the incidence of diabetes in 10 years with lifestyle interventions among people with impaired glucose tolerance [12]. Lifestyle changes during the postpartum period also affect anthropometry by reducing weight, BMI and waist circumference [13, 14] thus preventing the recurrence of GDM [15]. Xiang et al. [8] has confirmed that reducing insulin resistance either by a lifestyle or a pharmacological intervention will delay or prevent the development of T2DM in women with prior GDM.

Research on the impact of lifestyle interventions on mothers with a history of GDM is sparse in the South Asian region. Further, the findings of the western world are difficult to be adopted to lower middle income countries especially in the Asian region due to differences in socioeconomic status and cultural practices. Hence the aim of this study was to assess the impact of a simple, cost effective, culturally acceptable lifestyle intervention programme on the trajectory towards T2DM in women with a history of GDM.

## Materials and methods

### Study design and settings

A cluster randomized trial was conducted in 100 postpartum mothers with a history of GDM (Intervention group, *n* = 50; Controls, *n* = 50) in selected Medical Officer of Health (MOH) areas in three districts (Colombo, Gampaha and Galle) of Sri Lanka. Clinics were considered as clusters and the study was conducted in twelve clinics. Clusters were randomized by simple randomization to recruit patients to intervention and control groups.

### Study participants

Postpartum women with a history of GDM during the index pregnancy, diagnosed according to the standard guidelines [5] and who delivered a healthy singleton infant were recruited to the study. Those with a history of GDM in their previous pregnancies, a history of any chronic medical illness and those who are employed in the healthcare sector were excluded from the study.

### Recruitment of subjects

Postpartum women were recruited from five selected healthcare institutions in the three selected districts at the time of discharge from hospital after the delivery of the healthy singleton baby. After obtaining the informed written consent, the subjects were randomized to intervention and control groups based on the postpartum field clinics attended by them.

### Randomization

Mothers were invited from five purposively selected healthcare institutions in the three selected districts of Sri Lanka (Colombo, Gampaha and Galle), once they were discharged from the hospital. These mothers were attending twelve special postpartum clinics conducted in selected health unit areas of the three districts in the community setting. Clinics were considered as clusters and a cluster number was assigned to each clinic. Clusters were randomized to intervention and control groups based on the cluster number of the clinic by simple randomization, using a random number table.

### Procedure

The intervention programme was developed based on appraisal of literature and the findings of a previous qualitative study conducted by the same research team among the stakeholders [16] and opinions of the experts in the field. The protocol, consisting of dietary and exercise modifications, was designed to be administered individually, based on the BMI and the mode of delivery of each participant. This protocol was simple and cost-effective because it was a home-based protocol. They were educated to change their dietary and activity patterns without the effects on their routine practices or their day to day expenditure. This protocol was developed after considering their cultural values and beliefs [16] hence it was considered a culturally acceptable lifestyle intervention programme.

Each Participant in the intervention group was counselled for a minimum duration of 30 min when introducing the protocol. Although the details of the intervention are beyond the scope of this manuscript, restriction of carbohydrate intake was the key feature of the dietary intervention as we have already reported that these mothers have a tendency to consume a carbohydrate rich diet during the postpartum period due to the belief that carbohydrates enhance the breast milk production [16]. The unique feature in exercise recommendations was to guide the mothers to transform their day-to-day household work to energy expending activities targeting health benefits. The daily calorie requirement for mothers was based on their current BMI with minimal interference to breast feeding. Recommended daily intake for obese and overweight groups was 1800 kcal while mothers in the normal weight and underweight categories required 2300 kcal and 2425 kcal respectively.

Both intervention and control groups were requested to maintain a diet diary for three days (maintained on two week days and one day in the weekend) and an activity diary and pedometer recordings on seven days each month for the entire study period. Subjects in intervention groups were contacted once in two weeks by telephone to ensure adherence to the study protocol and subjects in control group contact to remind to maintain activity and diet diaries. The principal investigator visited the subjects in the intervention group once a month to reinforce the lifestyle intervention and to clarify any concerns regarding the protocol.

Both groups were followed up in follow-up clinics at 6 months and finally at one year. The glycemic parameters assessed were fasting and 2-hour post-OGTT plasma glucose, and HbA1c at baseline and after one year of intervention. The HOMA-IR was calculated using the values of fasting plasma insulin and fasting plasma glucose [17].

### Ethical approval and consent to participate

Ethical clearance was obtained from the Ethics Review Committee of the University of Sri Jayewardenepura (ERC 52/14), Sri Lanka and this study was registered in Sri Lanka Clinical Trials Registry (SLCTR/2015/021; date 25.09.2015). The trial was conducted adhering to the 1964 Declaration of Helsinki and its later amendments or comparable ethical standards. Subjects were invited to participate voluntarily for the study. They were informed of the aims, methods, benefits and possible discomforts verbally and through the information sheet provided to them. Informed written consent were obtained from the selected subjects for participation in the study.

### Analysis of dietary and physical activity data

Data obtained by the 3-day diet diary and the International physical activity questionnaire-(IPAQ)short version were analysed. The reliability of dietary data was ensured by verifying them with serial 24-hour dietary recalls. Physical activity data were validated by using the information of activity diaries and pedometer readings.

All foods recorded were converted into grams and the intake of total energy, macronutrients and selected micronutrients (calcium, iron and folate) were analysed and calculated by using Nutri Survey 2007 (EBISpro, Germany) which was modified for native food recipes based on food composition tables for Sri Lanka [18]. Physical activity levels were calculated in metabolic equivalent task minutes per week (MET-minutes/week) based on the IPAQ scoring protocol [19].

### Statistical analysis

Data were analysed using SPSS version 25.0 (IBM corporation, Armonk, NY). Socio-demographic characteristics, anthropometric measurements, dietary and physical activity data, and glycemic variables were described using descriptive statistics. Dietary data were log transformed when necessary and physical activity data were truncated to normalize the distribution.

Differences were considered significant if the P value was < 0.05. Normally distributed data were analysed using paired t -test. Skewed data were analysed using Kruskal-Wallis test. Logistic regression analysis was performed to control the effects of confounders. The factors significantly associated with insulin resistance were identified from previous literature and the univariate analysis performed. The variables that were significantly associated with the insulin resistance during the univariate analysis were incorporated in the regression model. A HOMA-ir of 2 or more [20, 21] was considered to define insulin resistance in this study.

## Results

### Socio demographic characteristics

Socio demographic characteristics of the subjects in the intervention and control groups are shown in Table 1. The mean age (SD) of the subjects in the intervention and control groups were 33.0 (5.1) and 34.3 (6.5) years respectively.

**Table 1:** Socio-demographic characteristics of the subjects in the intervention and control groups

Characteristic	Category	Intervention group (*n* = 50)	Control group (*n* = 50)	*p* value*
		*n* (%)	*n* (%)	
Age (years)	< 35	33 (66%)	26 (52%)	0.2
	≥ 35	17 (34%)	24 (48%)	
Monthly income (SL rupees)	< 50000.00	41 (82%)	34 (68%)	0.1
	≥ 50000.00	9 (18%)	16 (32%)	
Education	Up to GCE O/L	42 (84%)	34 (68%)	0.6
	GCE A/L and above	8 (16%)	16 (32%)	
Profession	Professional jobs	8 (16%)	9 (18%)	0.8
	Other jobs	14 (28%)	16 (32%)	
	Housewives	28 (56%)	25 (50%)	
Number of children	≤ 2	37 (74%)	38 (76%)	0.8
	> 2	13 (26%)	12(24%)	
Number of family members	< 5	22 (44%)	28 (56%)	0.2
	≥ 5	28 (56%)	22 (44%)	
Family history of DM	Positive	29 (58%)	26 (52%)	0.6
	Negative	21(42%)	24 (48%)	

GCE O/L - General Certificate of Education Ordinary level; GCE A/L – General Certificate of Education Advanced level p -value -Pearson chi square test

All sociodemographic characteristics were comparable between the intervention and control groups (*p* > 0.05) at baseline.

### Glycaemic parameters

Comparison of glycaemic parameters and insulin resistance within group and between group at baseline and 12 months are shown in Table 2.

**Table 2:** Comparison (within group and between group) of glycemic parameters and insulin resistance between baseline (6 weeks) and 12 months postpartum (mean + SD)

Outcome	Intervention	Control	Difference (p)
**FPG (mg/dl)**			
Baseline	89.6 (11.2)	91.9 (10.7)	2.3 (0.7)
12 months	87.3 (4.9)	123.2 (36.8)	28.4 (< 0.01)
Difference (p)	2.3 (0.0)	31.3 (0.1)	
**2-hour post-OGTT plasma glucose (mg/dl)**			
Baseline	109.2 (17.4)	113.4 (15.9)	4.2 (0.7)
12 months	106.5 (4.5)	156.1 (37.9)	50.5 (< 0.01)
Difference (p)	2.6 (< 0.01)	42.7 (< 0.01)	
**HbA1c (%)**			
Baseline	6.2 (1.6)	6.8 (2.2)	0.5 (0.07)
12 months	5.3 (0.4)	6.8 (1.9)	1.5 (< 0.01)
Difference	0.9 (0.0)	0.0 (0.8)	
**HOMA-ir**			
Baseline	1.9 (1.9)	2.5 (2.9)	0.6 (0.6)
12 months	0.9 (0.4)	2.3 (1.3)	0.2 (< 0.01)
Difference	1 (< 0.01)	0.6 (0.6)	

*p*-paired sample T test *p* > 0.05 (*p* = 0.05)

FPG, 2-hour post-OGTT plasma glucose, HbA1c(mean) and HOMA-ir were comparable between the two groups at baseline, which showed no significant difference between groups. However, all glycemic parameters showed a significant reduction in the intervention group compared to the control group at 12 months. The control group showed a significant increase in FPG, 2-hour post-OGTT plasma glucose and HbA1c at 12 months compared to baseline. Further, FPG, 2-hour post-OGTT plasma glucose (mean) and HbA1c remained within the normal recommended cutoffs in the intervention group while these parameters (mean) shifted to the prediabetes and diabetes levels in the control group [22].

**Fig. 1:**
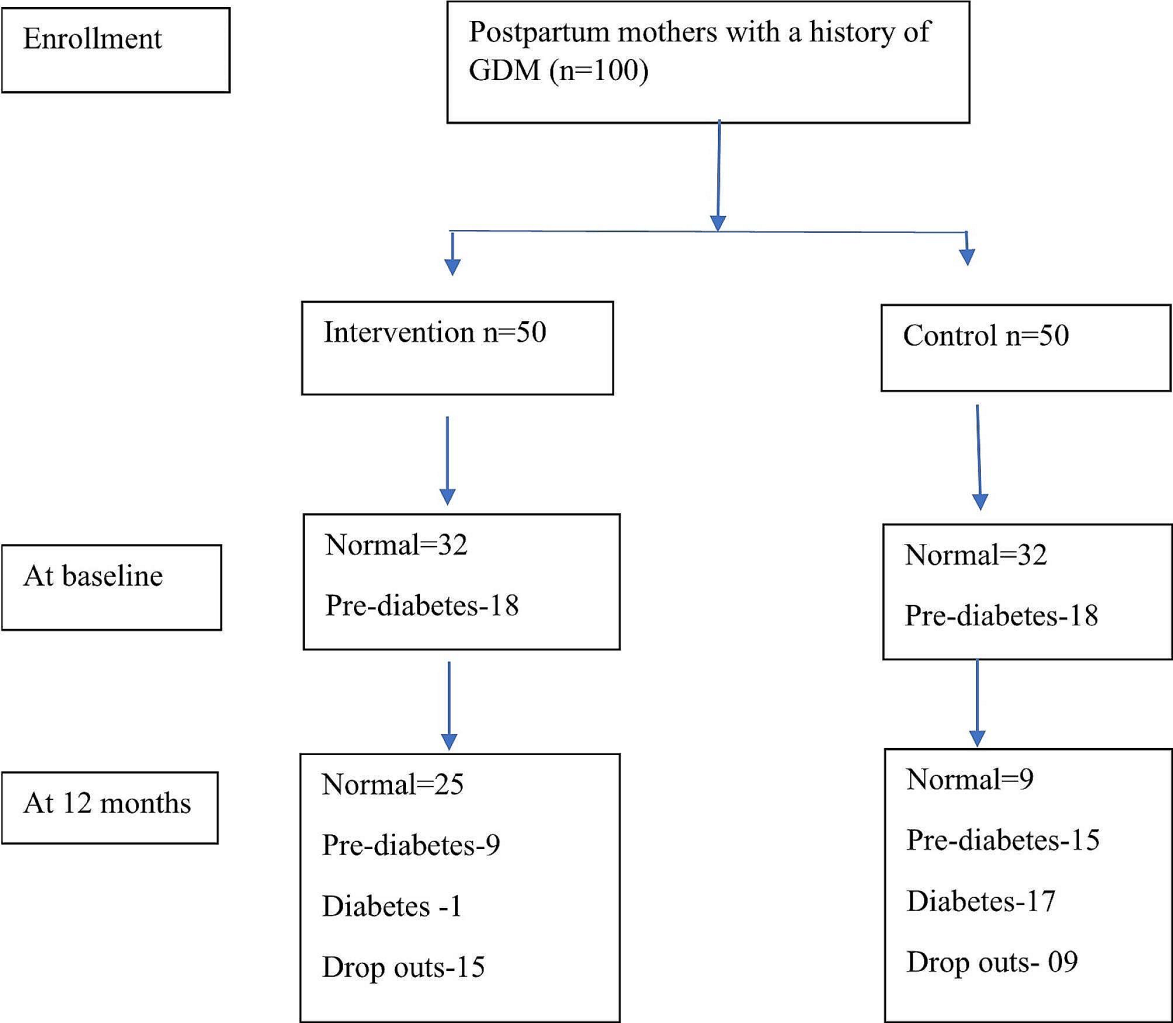
Consort flow diagram showing the outcome of all participants recruited for the study

Figure 1 is a flow diagram showing the outcome of all participants recruited for the study based on their HbA1C values.

During the first follow-up assessment at six months, four participants were removed from the intervention group while five participants were lost from the control group. Altogether four participants (two from the intervention and two from the control group) have changed their addresses and phone numbers without informing the research team. Three participants (two from the intervention and one from the control) were removed from the study due to non-compliance and two participants of control group due to high blood glucose.

At the 12th month altogether 21 participants (12 from intervention and nine from control group have been removed from the study). Together nine participants (seven from the intervention and two from the control group) have withdrawn from the study due to pregnancy. Altogether four participants (one from the intervention and two from the control group) withdrew due to high blood glucose values. One participant withdrew due to noncompliance with the intervention and one participant missed without informing from the intervention group. This information was presented in the consort diagram.

Logistic regression analysis Following factors were considered for the univariate analysis based on existing literature and expert opinion: parity, number of children, income, occupation, family history for diabetes mellitus, and whether they were in intervention group or control group. Only the factors which showed significant relationships with insulin resistance in the univariate analysis were considered for the model. The logistic regression analysis was performed to ascertain the effects of age, parity, family history of diabetes and the effect of intervention on the likelihood that the participants have on insulin resistance. The model explained 42% (Nagelkerke R ^2^) of the variance in insulin resistance and correctly classified 72.9% of cases (Table 3).

**Table 3:** Logistic regression analysis

Dependent variable	Independent variables	Standard Error	Adjusted Odd Ratio	Statistical significance	95% CI for Odd ratio	
					Lower	Upper
Insulin resistance	Control group	1.1	32.9	0.01	3.9	272.7
	parity	0.4	1.2	0.7	0.6	2.5
	Age	0.7	1.5	0.5	0.4	5.6
	Family history	0.7	1.6	0.5	0.4	5.7

In multiple linear regression model adjusted for age, parity and family history, the control group shows a 32.9 risk for insulin resistance compared to the intervention group.

### Anthropometric parameters

**Fig. 2:**
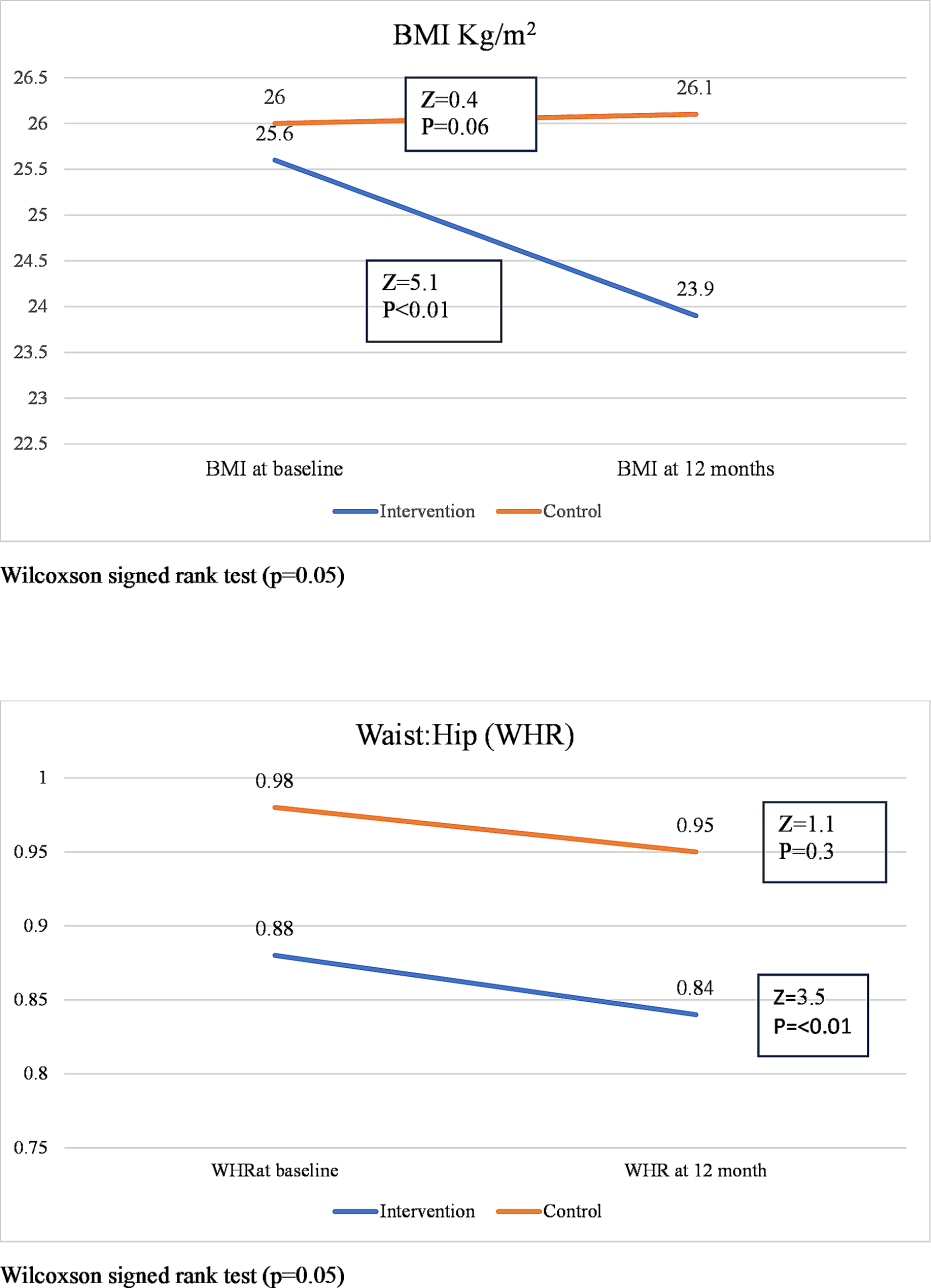
Changes in BMI and WHR (median) in the intervention and control groups at each time point studied

Changes in BMI and WHR (median) at 0 and 12-month time points in the intervention and control groups are shown in Fig. 2.

### The effect of the intervention on dietary and physical activity parameters

At the start of the intervention, both the energy intake (Intervention 2505.5 Kcal; Control 2535 Kcal, *p* = 0.59) and energy expenditure (Intervention 579.2 Kcal; Control 296.5 Kcal, *p* = 0.16) were comparable between the two groups. At the end of 12 months, the energy intake was significantly reduced in the intervention group (2031.2 Kcal with a difference of 474.3Kcal, p = < 0.001), whereas higher value was observed in the controls (3124.2 Kcal, *p* = 0.24). Although the energy expenditure was shown to reduce with the intervention (579.2 Kcal vs. 423.1 Kcal, *p* = 0.54), the change was not statistically significant. However, it was significantly higher than the energy expenditure in the control group (296.5 kcal vs. 271 Kcal, *p* = 0.61) at 12 months.

In the categorical analysis of physical activity data at 12 months, the number of mothers in high, moderate and low categories in the intervention vs. control groups were 25 vs. 10, 9 vs. 17 and 0 vs. 9 respectively, compared to as 29 vs. 23, 8 vs. 11 and 13 vs. 16 at baseline.

## Discussion

Type 2 diabetes mellitus is a rising threat throughout the world. Gestational diabetes mellitus is one of the major predisposing factors for the future type 2 diabetes mellitus [2]. Previous reports show that a culturally accepted lifestyle intervention can attenuate the progression of GDM to T2DM during the first postpartum year [23, 24]. However, since the postpartum period is a unique phase of life with its own concerns and limitations [25], the lifestyle change should be tailor made for optimum acceptance. Although GDM is a leading health care problem in the Asian region, available literature on postpartum lifestyle modifications for GDM mothers is very limited. This cluster randamized study designed to evaluate the effectiveness of a simple, cost effective and culturally acceptable lifestyle intervention programme developed to attenuate the progression of GDM to T2DM is an attempt to fill this gap. As postpartum women with comparable baseline characteristics were enrolled into the two groups by cluster randomization, the contamination of study populations was negligible.

The present study used a combination of fasting plasma glucose, 2-hour post-OGTT plasma glucose and HbA1c to determine the glycaemic status both pre and post intervention. The authors believe that the combination has improved the strength of our results. OGTT is considered as the gold standard diagnostic test which provides a sensitive and a specific diagnosis of abnormal carbohydrate metabolism in women with previous GDM compared to FPG alone or in combination with HbA1c [26]. HbA1c represents the average glucose level over the last three months. HbA1c in combination with OGTT would identify an additional 10.6% of pre-diabetic cases when used for screening of postpartum women with a history of GDM [27].

Glycaemic parameters are expected to return to normal immediately after childbirth in GDM women [6]. The findings of the present study were also in accordance with this as all women in both intervention and control groups had FPG and 2-hour post-OGTT plasma glucose values within normal limits at 6 weeks postpartum although HbA1C was slightly higher than the normal cut-off at 6 weeks. In this study, both fasting and 2-hour plasma glucose levels showed a significant decline from baseline to 12 months in the intervention group though both parameters were significantly increased in the control group with most normoglycemic subjects in the control group shifting to pre-diabetes and diabetes groups. However, another randomized controlled trial conducted on postpartum mothers with prior GDM in Australia failed to show an impact of the intervention on glycemic profile at 12 months postpartum [28]. In their study, although the intervention was introduced by one individual and five group sessions were held over a period of three months, only 10% of the participants of the intervention group had participated in all sessions. O’Really and the co-workers have stated difficulties in recruiting and delivering the intervention on postpartum women with young families [28]. The intervention in the current study was based on the results of a qualitative exploration of barriers and facilitators related to the postpartum period [16], which may have contributed to the success of the intervention.

HbA1c, which is another parameter used in this study is a good indicator of long term glycemic control [29]. According to our findings, the intervention group showed a significant lowering of HbA1c at 12 months compared to baseline which was not seen in the control group. Further, on looking at the outcome of all participants at the end of the study period, only one subject (2%) in the intervention group has developed T2DM, when compared to a much higher proportion of 34% among the controls. This observation clearly denotes a better glycemic regulation in the intervention group compared to the controls which is likely to be due to the intervention. Furthermore, the results enable us to postulate the satisfactory adhesion of the subjects to the intervention. However, according to the final assessment based on HbA1c values, there were nine participants in prediabetes and one participant in the diabetes category in the intervention group. Some factors which were beyond the scope of this study i.e. genetic factors and epigenetic modifications have also been found to be associated with the development of GDM and DM. In this study, we did not explore these relationships and these may be the reasons for the observed negative results.

Pregnancy-related insulin resistance returns to the pre-pregnancy status immediately after the delivery of the placenta [30]. Increased levels of fasting insulin increase the risk of pre-diabetes in women [31] and physical activity is known to enhance the maternal insulin sensitivity thereby decreasing the need for insulin [32, 33]. HOMA–ir which denotes insulin resistance has decreased in the intervention group from baseline to 12 months with a statistically significant difference, whereas the change was not significant in the controls. This study supports a previous observation of the reduction of fasting insulin induced by a dietary and exercise intervention targeting a change in weight [34]. Moreover, the observation of a significantly reduced HOMA-ir in the intervention group compared to controls further strengthens the value of the lifestyle intervention. Findings of the present study are also in line with a randomized controlled trial involving Jewish and Bedouin post-GDM women in Israel [24]. The unique feature shared by both studies is that they were developed considering the cultural norms of the populations concerned, to enhance the acceptability of the intervention. The results of the present study were further strengthened by the logistic regression model which confirmed that the controls were 33% more likely to show insulin resistance compared to the women in the intervention group after controlling for the other risk factors [35–37] that contribute to the development of T2DM. The intention-to-treat analysis helps to handle missing data in randomized controlled trials [38]. In our study, this method was used to handle the missing data to enhance the quality of data. In this study, substituting the missing data with sample median.

The finding of a significant reduction of BMI and WHR parameters at 12 months postpartum in the intervention group compared to the controls is good evidence of achieving the objectives of the intervention programme. It is also interesting to see how these parameters have changed in the two groups over the period of 12 months. Both BMI and the WHR showed a significant decline over the 12-month period in the intervention group while they remained almost unchanged in the control group. Aligning with our results, in a study of postpartum Swedish women, Huseinovic et al. (2016) [39] have reported a 3.4 kg/m2 (median) reduction in BMI following the intervention. Although the intervention of the present study was somewhat similar, the reduction of BMI achieved was much lower (1.71 kg/m^2^) probably because the lifestyle intervention offered by the Swedish investigators was more target oriented with weekly and final weight loss goals. In a randomized controlled trial in which the lifestyle modifications were administered via a web-based programme, a mean weight loss of 2.8 kg and a mean weight gain of 0.5 kg compared to baseline were observed at 12 months postpartum in the intervention and control groups respectively [40].

The objective of the lifestyle intervention in the present study was to reduce the energy intake and enhance the energy expenditure. As per the expectations, there was a significant reduction of the energy intake in the intervention group compared to controls at 12 months postpartum. This finding gives an indirect indication of the feasibility of the proposed dietary intervention as it is likely that these women have adhered to the recommendations. Although the targeted increase in the energy expenditure between baseline and 12 months was not achieved with the intervention, the energy expenditure was significantly higher in the intervention group when compared to the controls at the end of the study period and majority of mothers in the intervention group were in high or moderately active categories.

Cultural sensitivity of the intervention was the major strength of the present study as the dietary and physical activity modifications were based on the perceptions of the postpartum women [16]. Majority of the participants could achieve the goals of the intervention due to the continued follow-up, guidance and support. Although self-reported dietary and physical activity data (24-hr dietary recall and the activity diary) were used for analysis, the concurrent use of a 3-day diet diary and IPAQ compensated for the recall bias to a great extent. Since the postpartum women belong to a group with a unique set of problems, regular reinforcement of the intervention is mandatory for optimum adherence. This study used motivational telephone calls once in two weeks to ensure optimum adherence of participants to the protocol.

Lack of blinding was identified as a potential bias in this study. However, researchers took measures as much as possible to reduce the impact of this bias. Almost all the outcomes were measured objectively. Blood investigations were done in an accredited laboratory without involvement of the research team members. Physical activities were also measured by an objective tool (the pedometer).

The feasibility of the intervention was checked by conducting focus group discussions and in-depth interviews with stakeholders after completion of one year of the intervention programme. However, due to financial constraints and time barriers, research team members could not observe the long-term impact of these lifestyle interventions.

There are nine provinces in Sri Lanka. We selected two provinces from out of nine to select the three districts. These two provinces also represent the majority of postpartum mothers in the country since they share similar cultural characteristics and traditional beliefs and practices. Further, these two provinces represent urban, rural, and semi-urban populations. However, we would like to recommend replicating this study in different cultural backgrounds in Sri Lanka to enhance the generalizability of the findings.

## Conclusions

A culturally acceptable, individualized and well monitored lifestyle intervention comprised of dietary and physical activity modifications implemented in the community setting was able to produce remarkable reductions in glycaemic parameters by reducing insulin resistance among postpartum women with a history of GDM.

## Data Availability

The datasets used and analysed during the current study available from the corresponding author on reasonable request.
